# Interpersonal Predictors of Youth Internalizing Symptom Trajectories During the COVID‐19 Pandemic

**DOI:** 10.1002/jad.70245

**Published:** 2026-08-04

**Authors:** Nicole S. J. Dryburgh, Ryan Miller, Eric Duku, Katherine T. Cost, Yun‐Ju (Claire) Chen, Jordan Edwards, Emma Nolan, Katholiki Georgiades

**Affiliations:** ^1^ Offord Centre for Child Studies McMaster University Hamilton Ontario Canada; ^2^ Department of Psychiatry and Behavioural Neurosciences McMaster University Hamilton Ontario Canada; ^3^ Department of Psychology Harvard University Cambridge Massachusetts USA; ^4^ Department of Psychology University of Waterloo Waterloo Ontario Canada; ^5^ Department of Occupational Therapy, Graduate Institute of Behavioral Sciences Chang Gung University Taoyuan Taiwan; ^6^ Department of Psychiatry Chang Gung Memorial Hospital at Linkou Taoyuan Taiwan; ^7^ Hamilton Health Sciences Hamilton Ontario Canada; ^8^ Department of Psychology Queen's University Belfast Belfast Ireland

**Keywords:** COVID‐19, longitudinal, pandemic, relationships, youth mental health

## Abstract

**Introduction:**

The COVID‐19 pandemic disrupted youths' interpersonal relationships, posing risks for internalizing distress. Yet, evidence remains limited beyond the early pandemic waves and with changing interpersonal contexts. This study examined trajectories of youth internalizing symptoms across five timepoints of the pandemic and associations with parental support and social interactions (in‐person vs. virtual).

**Method:**

Data were from the population‐based COVID‐19 Ontario Provincial Assessment and Tracking of Child and Family Health study in Canada. Youth (*N* = 176; 10–18 years; 51.4% girls, 43.5% boys) participated in up to five waves between January 2021 and December 2022. Linear mixed‐effects models were conducted with internalizing symptoms as the outcome and age, gender, parental support, and in‐person and virtual social interactions as predictors.

**Results:**

Girls (*b* = 0.21, *p* = 0.005), gender minority youth (*b* = 0.60, *p* < 0.001), and older youth (*b* = 0.07, *p* < 0.001) reported greater symptoms; girls showed steeper increases over time (*b* = 0.01, *p* = 0.006), reflecting widening gender disparities. Greater baseline parental support was associated with fewer symptoms (*b* = −0.33, *p* < 0.001), with a stable effect over time. In‐person and virtual interactions were unrelated to symptoms at baseline; however, over time, more frequent in‐person interactions were increasingly associated with fewer symptoms (*b* = −0.01, *p* = 0.005). Virtual interactions remained unrelated.

**Conclusions:**

Internalizing symptoms increased modestly across the study period, with widening disparities for girls that extended well beyond periods of lockdown. In‐person social interactions became increasingly protective, whereas parental support showed a stable protective association. Findings highlight the importance of gender‐responsive supports and family relationships during prolonged disruption, and in‐person connections as restriction ease.

The COVID‐19 pandemic presented substantial difficulties for children and youth globally. Repeated school closures, reduced access to services, canceled extracurricular activities, and prolonged restrictions on social contact created conditions that are well‐documented to heighten risk for anxiety and depression (Mazrekaj and De Witte 2024). Early evidence documented sharp increases in these symptoms among youth during the initial months of the pandemic (Geoffroy et al. 2024; Madigan et al. 2023; Racine et al. 2021). However, less is known about whether these increases persisted, improved, or worsened as the pandemic evolved, and whether trajectories varied according to interpersonal relationships.

Of the Group of 10 (G10) countries, Canada implemented some of the most restrictive measures to curb the COVID‐19 virus, including repeated school closures, likely impacting youths' adjustment and relationships (Razak et al. 2022). A meta‐analysis of Canadian evidence suggested a slight worsening of depressive symptoms for youth at pandemic onset (first wave defined here as March–August 2020), followed by a return to pre‐pandemic levels by the second wave (September 2020–mid‐February 2021), though anxiety symptoms—particularly among girls—appeared to worsen from the first to third wave (late February–July 2021; Geoffroy et al. 2024; Wu et al. 2021). However, few longitudinal studies extended beyond mid‐2021, despite subsequent continued waves (fourth: August–December 2021; fifth: December 2021–February 2022) (Felix and Green 2025; Geoffroy et al. 2024; Ontario COVID‐19 Hospital Admissions and Deaths by Age from Waves 1‐7 2023). Many studies relied on convenience or clinical samples, limiting generalizability (Geoffroy et al. 2024). As a result, the longer‐term course of youth internalizing symptoms across later pandemic phases remains unclear, as does the question of *for whom* these difficulties persisted. Understanding these longer‐term trajectories is critical for informing prevention efforts and preparedness for youth transitioning to post‐pandemic contexts.

Supportive relationships with parents/caregivers are consistently associated with positive youth adjustment and may buffer against psychological difficulties (Keijser et al. 2020; Rueger et al. 2016; Smokowski et al. 2015). Studies conducted early in the pandemic—primarily outside of Canada—suggested that greater parental support was associated with fewer internalizing concerns (Campione‐Barr et al. 2025; Gawrych et al. 2023). However, a recent meta‐analysis identified only one study examining social support and the mental health of Canadian youth during the pandemic (Park et al. 2023; Zhang et al. 2025). This study found that parent‐reported social support (i.e., a combination of support from both family and peers) was not independently associated with the child's future depressive symptoms over and above the parents' report of the child's social connectedness. Importantly, the study involved a predominantly clinical sample and data were available only through early 2021. It remains unclear whether parental support was protective for youth in the general population, and whether this potential protective role persisted as the pandemic evolved, specifically, at later stages when youth regained opportunities for social contacts outside of home.

Peer relationships also play a critical role in socioemotional adjustment during adolescence (Rubin et al. 2015; Bagwell and Bukowski 2018). Disruptions to in‐person interactions raised concerns about longer‐term consequences for youth mental health (Vaillancourt et al. 2021). Indeed, early in the pandemic, youth expressed concerns about reduced time with friends and family with whom they did not live, and reduced contact with friends was associated with greater loneliness and internalizing symptoms (Ellis et al. 2020; Rodman et al. 2024; Silk et al. 2022). Many youth substituted face‐to‐face contact with virtual interactions, such as phone or video calls. Some early studies suggested that virtual contact buffered against loneliness and distress (Marciano et al. 2022), whereas others emphasized the unique benefits of in‐person interactions for affective co‐regulation and belonging (Achterhof et al. 2022; Ellis et al. 2020). Whether in‐person and virtual interactions were differentially associated with internalizing difficulties during later pandemic phases, and whether these associations changed over time, remains unclear. Examining potential buffering effects is also needed to inform future pandemic efforts. For example, in‐person and virtual social interactions may have been more strongly associated with fewer internalizing difficulties for youth with lower parental support.

## 1 Objectives

The current study provides new evidence using longitudinal, population‐based data from Ontario youth collected between January 2021 and December 2022. The objectives were to examine (1) changes in youth internalizing symptoms beyond the initial phases of the pandemic; (2) whether symptom trajectories differed by gender, age, or baseline parental support; and (3) whether the frequency and type of social interactions (in‐person vs. virtual) were associated with symptom levels or trajectories across time. We hypothesized that internalizing symptoms would be heightened during times of restrictions and lower as restrictions eased. We also hypothesized that greater parental support at baseline would be associated with fewer symptoms, and that more frequent in‐person and virtual interactions would be protective, particularly for youth reporting lower parental support. We again examined whether these effects differed with time.

## 2 Methods

### 2.1 Study Design and Participants

Data were drawn from the COVID‐19 Ontario Provincial Assessment and Tracking of Child and Family Health (ONPATH) study, a population‐based longitudinal study of children and youth during the pandemic (Georgiades et al. 2026). Five waves of data were collected between January 2021 and December 2022, approximately every 4–6 months, timed to maximize variation in public‐health COVID‐19 restrictions. Baseline data were collected during partial lockdown, followed by a province‐wide lockdown (Time 2), partial reopening (Times 3–4), and full reopening (Time 5). Full details including the sampling frame are available in preprint (Georgiades et al. 2026).

### 2.2 Sample for Analysis

The analytic sample included youth who participated at baseline and at least one additional wave (*N* = 176, *M*_*age*_ = 14.53, *range* = 10–18, SD = 2.07; 77.0% of full sample). Youth participated in two to five waves (*M* = 3.1; see Table 1). The analytic sample did not differ from the full sample on any demographic characteristics (see Supporting Information S1: Table 1), and included 51.1% cisgender girls, 43.8% cisgender boys, 4.5% gender minority youth, and 0.6% who reported different gender identities over time (see Measures).

**Table 1:** Descriptive statistics of sample and main study variables.

Timepoint	Mean (SD) Internalizing (min = 0, max = 2)	Mean (SD) In‐Person Interactions (min = 0, max = 2)	Mean (SD) Phone/Video Interactions (min = 0, max = 2)
Baseline	0.75 (0.54)	0.38 (0.63)	0.92 (0.81)
Timepoint 2	0.80 (0.57)	0.49 (0.72)	0.80 (0.74)
Timepoint 3	0.71 (0.51)	0.88 (0.81)	0.62 (0.64)
Timepoint 4	0.84 (0.56)	0.87 (0.85)	0.71 (0.68)
Timepoint 5	0.81 (0.63)	0.98 (0.83)	0.60 (0.69)

### 2.3 Procedure

This study was approved by the Hamilton Integrated Research Ethics Board (HiREB Project #: 10855). All procedures were available in English and French and were the same across all waves. Participants were contacted by email and invited to participate. For those who did not respond, follow‐up calls were conducted by trained bilingual interviews by telephone. Participants were asked to provide consent by online form or through documented verbal consent and were able to choose to complete the surveys online or by telephone. In households with more than one eligible child, the selected child was the one who had their most recent birthday at the time of the baseline survey. Parents of youth aged 11 or older were asked for consent to contact their child and invite them to complete the youth survey. All study materials were available in English and French. Participants were entered into a lottery draw for three prizes ($200 each) at each wave of data collection.

### 2.4 Measures

#### 2.4.1 Internalizing Symptoms

The primary outcome was internalizing symptoms as assessed by an abbreviated version of the Ontario Child Health Study Emotional Behavioural Scales (OCHS‐EBS; Duncan et al. 2019), a validated measure that assesses youths' mental health symptoms. The abbreviated version was 21 items with evidence of excellent internal consistency (all subscales *α* > 0.9) (Georgiades et al. 2026). The scale includes symptoms of Major Depressive (e.g., “I feel worthless or inferior”) and Generalized Anxiety Disorders (e.g., “I am fearful or anxious”). Youth rated how much each item described them in the past week from 0 = “*never or not true*” to 2 = “*often or very true*.” As few data were missing (maximum 0.7%), mean scores were computed.

#### 2.4.2 Parental Support at Baseline

Youth rated five items about support from their parent(s) or caregiver(s) (i.e., can talk to about problems, trust and rely on for support, listens to me, really cares about me, and believes I will be a success) on a 4‐point scale from 0 = “*strongly disagree*” to 3 = “*strongly agree*.” As there were minimal missing data (maximum 2.6%), responses were averaged.

#### 2.4.3 Frequency of In‐Person and Virtual Social Interactions

Youth reported on how often they interacted in person or by phone or video calls with friends or family with whom they did not live in the past week at each timepoint. Response options for in‐person interactions ranged from 0 = “*never*” to 5 = “*every day*.” Based on the distribution of responses, responses were collapsed into three categories, specifically: low = *less than or equal to 1 day*, moderate = *2–3 days*, and high = *4 days or more*. Response options for phone/video calls were: 0 = “*less than 1 h*,” 2 = “*1–2 h*,” 3 = “*2–3 h*,” 4 = “*3–4 h*,” 5 = “*4 h or more*.” Responses were recoded into low = *less than 1 h*, moderate = *1–4 h*, and high = *4 h or more*.

#### 2.4.4 Time

Time was coded as 0 for the baseline assessment. For all other timepoints, time was represented by the number of months since the baseline survey.

#### 2.4.5 Youth Age at Baseline

Age was calculated from youth reports of their birth year and month at the first timepoint.

#### 2.4.6 Youth Gender

Gender was assessed with youth‐report when available, and parent report when not by asking “What is your/your child's gender? Gender refers to current gender which may be different from sex assigned at birth and may be different from what is indicated on legal documents” with female, male, another gender (specify), and prefer not to answer as response options. Open‐ended responses from those who selected “another gender” included agender, fluid, non‐binary, and listed pronouns (e.g., she/they). As the proportion of youth endorsing each gender minority identity was too few for comparative analyses, and to retain these youth in models, a three‐category gender variable was used in all analyses (i.e., boy = 0, girl = 1, and gender minority identity = 2). Of note, one youth's report of their gender identity changed throughout data collections and was sometimes missing. Based on recent recommendations regarding missing gender identity data (Soneson et al. 2024) we included this participant under the gender minority identity category.

### 2.5 Analytic Plan

To examine changes in internalizing symptoms, in‐person interactions, and virtual interactions over the study period, descriptive statistics were first examined for each timepoint. An unconditional model was examined with the inclusion of a random intercept to account for clustering of data within participants, and the intraclass correlation coefficient (ICC) calculated to identify sources of variation in internalizing symptoms—that is, between‐individual (Level 2) differences versus within individuals (Level 1) across time. Polynomial time contrasts and visual inspection of diagnostic plots were used to test for patterns (i.e., linear, quadratic, cubic) of change. Predictors of change in internalizing symptoms were examined using a three‐phase modeling approach. All models included random intercepts to account for clustering within participants. First, time and demographic characteristics (age and gender, with boys as the reference group) were added as predictors. Second, parental support at baseline (grand mean centered) was added as a time‐invariant predictor. Third, the frequency of social interactions—both in‐person and virtual—at each timepoint were added as time‐varying predictors. We first added main effects of each predictor, followed by interaction terms with time to test whether the associations between each predictor and internalizing symptoms varied with time. We performed post‐hoc probing of significant interactions using estimated marginal means for categorical moderators and simple slopes and Johnson‐Neyman analyses for continuous moderators and plotted model‐based predicted values for interpretation. Finally, we tested three‐way interactions to examine whether any significant time‐varying effects of in‐person or virtual interactions differed depending on baseline parental support. Model comparisons were performed using likelihood ratio tests, with models fitted using maximum likelihood estimation to ensure valid comparison of fixed‐effects structures. Analyses were conducted using the *lme4* package in R.

## 3 Results

### 3.1 Changes in Youth‐Reported Mental Health Symptoms Across Time

Descriptive statistics for internalizing symptoms and the frequency of in‐person and virtual interactions at each timepoint are presented in Table 1. Based on the observed values, mean internalizing symptom scores ranged from 0.71 to 0.84, with the highest mean observed at the second (*M* = 0.80, SD = 0.57) and fourth timepoints (*M* = 0.84, SD = 0.56)—times representing a period of province‐wide lockdown and shortly after a period of increased restrictions due to the Omicron variant, respectively.^1^ 1 Mean in‐person interactions appeared to increase with each timepoint. Virtual interactions appeared to decrease in frequency with each timepoint, with the exception of a small increase at the fourth timepoint (*M* = 0.71, SD = 0.68).

An unconditional (null) random‐intercept model was fit to partition variance in internalizing symptoms into within‐ and between‐person components. The model indicated substantial between‐person variability in internalizing symptoms (*σ* ^*2*^ = 0.22, SD = 0.47) relative to within‐person variability (*σ* ^*2*^ = 0.09, SD = 0.30). The intraclass correlation coefficient (ICC) indicated that approximately 70% of the variance in internalizing symptoms was attributable to between‐individual differences (ICC = 0.70) and about 30% of the variance was attributable to differences within individuals across time.

### 3.2 Time, Demographics, and Interpersonal Predictors of Internalizing Symptoms

The results of the iterative linear mixed‐effects models examining time, demographics, and interpersonal factors as predictors of internalizing symptoms are presented in Table 2. Model 1 examined time as a predictor of internalizing symptoms, which indicated a very small but significant positive association between time since study start and internalizing symptoms, *b* = 0.004, *p* = 0.03. Polynomial contrasts treating time as a categorical factor did not detect significant nonlinear trends (quadratic: *t*(401.24) = 0.10, *p* = 0.92; cubic: *t*(402.37) = −0.11, *p* = 0.91), suggesting that change was gradual rather than characterized by distinct shifts between assessment waves or other patterns of change. Together, these findings indicated a small overall increase in internalizing symptoms over the study period, with substantial between‐ and within‐person differences across time.

**Table 2:** Results of linear mixed models testing predictors of youth‐reported internalizing symptoms.

	Model 1: Time				Model 2: Demographics				Model 3: Interpersonal			
Predictors	*b*	*CI*	*t*	*p*	*b*	*CI*	*t*	*p*	*b*	*CI*	*t*	*p*
(Intercept)	0.74	0.67–0.82	18.74	< 0.001	0.61	0.51–0.72	11.26	**< 0.001**	0.59	0.47–0.70	9.71	**< 0.001**
Time (months)	0.004	0.00–0.01	2.12	0.03	−0.002	−0.01 to 0.01	−0.70	0.48	0.003	−0.01 to 0.01	0.73	0.46
Girl					0.21	0.06–0.35	2.80	**0.005**	0.20	0.07–0.33	2.98	**0.003**
Gender Minority Identity					0.60	0.27–0.94	3.58	**< 0.001**	0.52	0.22–0.83	3.40	**< 0.001**
Age at baseline (Centered Between)					0.07	0.04–0.11	4.23	**< 0.001**	0.06	0.03 – 0.09	3.98	**< 0.001**
Time X Girl					0.01	0–0.02	2.77	**0.006**	0.01	0.00–0.02	2.26	**0.02**
Time X Gender Minority Identity					0.001	−0.02 to 0.02	0.12	0.90	0.001	−0.02 to 0.02	0.09	0.93
Time X Age					−0.001	0–0	−1.24	0.22	−0.001	0–0	−1.05	0.30
Parental Support									−0.33	−0.46 to −0.21	−5.28	**< 0.001**
Time X Parental Support									0	−0.01 to 0.01	−0.06	0.95
In‐Person Interactions									0.03	−0.04 to 0.09	0.87	0.38
Virtual Interactions									0.02	−0.04 to 0.08	0.54	0.59
Time X In‐Person Interactions									−0.01	−0.01 to −0.002	−2.81	**0.005**
Time X Virtual Interactions									0.004	−0.002 to 0.01	1.28	0.20
Parental Support X In‐Person Interactions									−0.05	−0.18 to 0.07	−0.82	0.41
Time X Parental Support X In‐Person Interactions									0.003	−0.01 to 0.01	0.50	0.62
Model fit *χ2* (1)	4.45, *p* = 0.03				49.11, *p* < 0.001				51.79, *p* < 0.001			
σ^2^	0.09				0.09				0.09			
τ_00 id_	0.22				0.17				0.13			
ICC	0.70				0.65				0.59			

Model 2 added the demographic predictors age and gender (girls and gender minority youth) and interaction terms with time. Findings indicated greater internalizing symptoms for girls compared to boys (*b* = 0.21, *p* = 0.005), as well as a significant time by gender interaction for girls compared to boys (*b* = 0.01, *p* = 0.006). This interaction is displayed in Figure 1. Post‐hoc comparisons indicated that girls reported significantly higher internalizing symptoms than boys at all time points: At baseline, girls scored higher than did boys (*b* = 0.21, *p* = 0.006), and this difference increased over time (*Time 2: b* = 0.27, *p* < 0.001; *Time 3: b* = 0.32, *p* < 0.001; *Time 4: b* = 0.37, *p* < 0.001; *Time 5: b* = 0.44, *p* < 0.001), indicating that the gender disparity in internalizing symptoms widened across the study period. To further examine the trajectory of internalizing symptoms among girls, a post‐hoc analysis was conducted in which time was modeled as a categorical variable for the subgroup of girls only. The likelihood ratio test indicated significant differences in internalizing symptoms for girls across waves, *χ* ^*2*^(4) = 17.54, *p* = 0.002. Tukey‐adjusted pairwise comparisons indicated that girls' internalizing symptoms were significantly greater at Time 4 (*mean difference* = 0.20, *p* = 0.008) and Time 5 (*mean difference* = 0.21, *p* = 0.016) compared to Time 1. No other significant differences emerged, including between Time 4 and Time 5 (mean difference = 0.01, *p* = 0.99), suggesting that internalizing symptom levels increased from the early pandemic period and remained elevated through the final study wave for girls.

**Figure 1:**
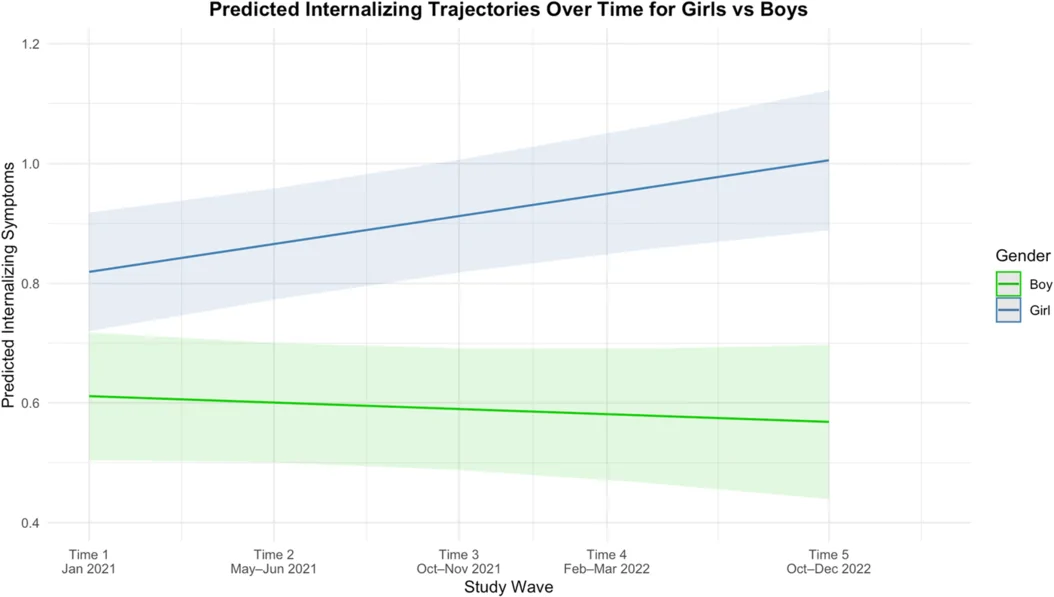
Predicted internalizing symptom trajectories over time as a function of gender (girls vs. boys). Predicted values were derived from the mixed‐effects model. Shaded ribbons represent ± 1 SE around predicted values; analyses control for age.

Symptoms were also greater for gender minority youth compared to boys (*b* = 0.60, *p* < 0.001), and for older youth compared to younger youth (*b* = 0.07, *p* < 0.001). Neither of the interactions for time and age nor time and gender minority youth were significant (time by age: *b* = −0.001, *p* = 0.22; time by gender minority youth: *b* = 0.001, *p* = 0.90).

Model 3 added interpersonal predictors: baseline parent support, in‐person social interactions, and virtual interactions, and interaction terms between each predictor and time. Results indicated that greater parental support at the start of the study was associated with fewer internalizing symptoms (*b* = −0.33, *p* < 0.001). The interaction term between parental support and time was not significant (*b* = 0, *p* = 0.95), suggesting that the association between baseline parental support and internalizing symptoms was consistent across the study period. In‐person (*b* = 0.03, *p* = 0.38) and virtual social interactions (*b* = 0.02, *p* = 0.59) were unrelated to internalizing symptoms, but there was a significant interaction between in‐person interactions and time (*b* = ‐0.01, *p* = 0.005). Post‐hoc simple slope and Johnson‐Neyman analyses were performed, and the model‐based predicted values are displayed in Figure 2.

**Figure 2:**
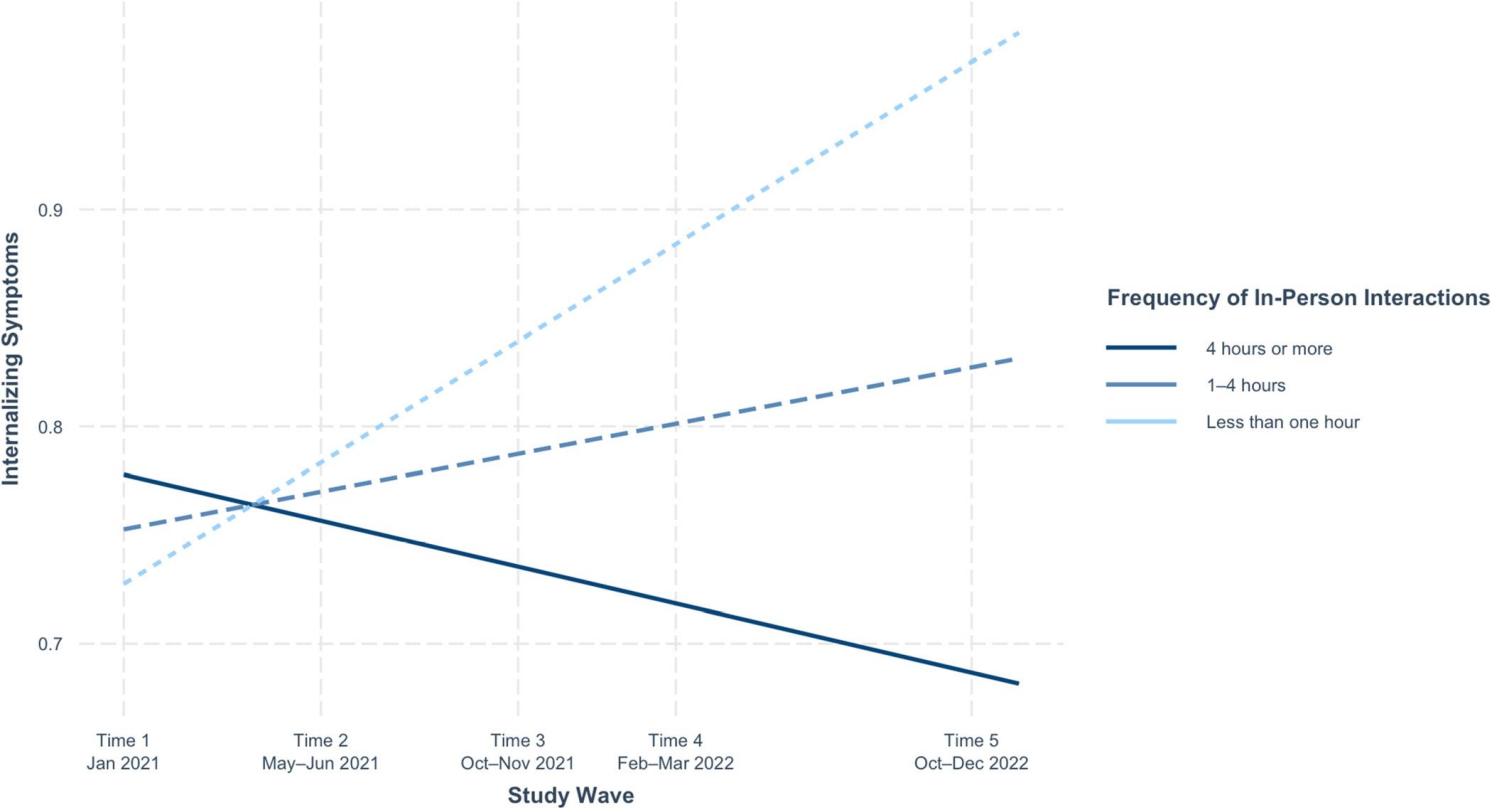
Predicted internalizing symptom trajectories over time as a function of in‐person interaction frequency. Predicted values were derived from the mixed‐effects model. Analyses control for sex, age, gender identity, parental support, and virtual interactions.

As displayed, at baseline, frequency of in‐person interactions was unrelated to internalizing symptoms; however, over time, greater in‐person interaction was increasingly associated with fewer symptoms. In contrast, the interaction between time and virtual interactions was nonsignificant (*b* = 0.004, *p* = 0.20), indicating that the association between virtual contact and internalizing symptoms did not differ across time. A 3‐way interaction testing whether the time‐varying effect of in‐person interaction differed with parental support was not significant (*b* = 0.003, *p* = 0.62), suggesting that the protective trajectory associated with in‐person contact did not significantly differ by initial levels of parental support.

Exploratory analyses examined whether associations involving parental support, in‐person social interactions, and virtual social interactions differed by gender. No moderation effects were observed for parental support or virtual social interactions (all *p*s > 0.28), indicating that associations involving these predictors were comparable across gender groups. However, a significant interaction emerged between gender (cisgender girl vs. cisgender boy) and in‐person social interactions (*b* = 0.12, *p* = 0.007). Follow‐up analyses indicated that more frequent in‐person social interactions were associated with lower internalizing symptoms among boys, whereas this association was not evident among girls. No differences were observed between gender minority youth and boys (*p* = 0.47). Finally, adding higher‐order Gender X Social Interaction X Time interactions did not significantly improve model fit, *Δχ* ^2^(6) = 8.20, *p* = 0.22, suggesting that the longitudinal associations between social interactions and internalizing symptom trajectories were consistent across genders.

## 4 Discussion

This study provides new longitudinal evidence on youth internalizing symptoms across multiple phases of the COVID‐19 pandemic. This evidence is provided by leveraging a population‐based sample in Ontario, Canada—a province that implemented some of the most stringent public health measures at this uncertain time (Razak et al. 2022). Three primary findings emerged. First, increases in internalizing symptoms were observed over time, but this pattern was specific to girls. Girls reported greater symptoms at baseline, as did older youth. Girls further exhibited a more adverse symptom trajectory than did boys across the study period, with significantly higher internalizing symptoms at Time 4 and Time 5 than at the initial timepoint, with symptom levels remaining elevated through the final study wave. Internalizing symptoms did not differ significantly among the first three study waves (Times 1–3), suggesting that the observed increase emerged later in the study period rather than immediately following the initial lockdown phases. These findings suggest that girls experienced persistently elevated internalizing symptoms beyond the most stringent lockdown phases into periods when public health restrictions were easing. The overall effect of time was not significant once gender was included, and no significant change was observed for boys. These findings are consistent with documented gender disparities in adolescent internalizing distress during the pandemic, including in Canadian contexts (Geoffroy et al. 2024; Madigan et al. 2023; Racine et al. 2021), and extend this evidence later into the pandemic period. Second, parental support showed a robust association with lower youth internalizing symptoms, underscoring the importance of supportive parent‐child relationships during prolonged periods of disruption. Third, the protective effects of in‐person social interactions strengthened over time, as opportunities for face‐to‐face contact increased with the easing of restrictions. In contrast, virtual interactions were unrelated to internalizing symptoms. Together, these findings highlight the continued vulnerability of girls to elevated internalizing symptoms over the course of the pandemic, alongside the importance of both family support and access to in‐person peer connections for youth emotional adjustment.

The small mean change in internalizing symptoms over time that was accounted for by demographic factors, coupled with large between‐person variability for youth, aligns with prior research from early in the pandemic indicating heterogeneous mental‐health responses among youth (e.g., Cost et al. 2022; Racine et al. 2021). For example, a study of adolescents across three waves early in the pandemic (November 2020, February 2021, May 2021) found similar results, such that 25%–30% of the variation in internalizing symptoms was attributable to within‐individual differences over time (Liang et al. 2024).

Consistent with prior Canadian and international studies, girls reported greater internalizing symptoms at baseline and exhibited steeper symptom trajectories than did boys (Geoffroy et al. 2024). Post‐hoc analyses indicated that girls' internalizing symptoms did not differ significantly across the first three study waves (Times 1–3). In contrast, symptom levels at Times 4 and 5 were significantly higher than at Time 1. Symptom levels did not differ between Times 4 and 5, suggesting that increases for girls emerged later in the study period and remained elevated through the final wave. These findings align with both pre‐pandemic and pandemic‐era evidence documenting greater vulnerability to internalizing difficulties among adolescent girls, theorized to be driven by a combination of intersecting biological, cognitive, and social‐contextual factors (Geoffroy et al. 2024; Halldorsdottir et al. 2021). These disparities may have been widened by pandemic‐related stressors that disproportionately affected girls, such as disruptions to peer relationships, increased caregiving demands, or heightened academic and social pressures (Hutchinson et al. 2021), highlighting the need for gender‐responsive prevention efforts during periods of widespread societal stress. Although girls reported higher internalizing symptoms and steeper increases over time than boys, exploratory moderation analyses suggested that the predictors examined generally operated similarly across gender groups. Specifically, we found no evidence that the associations of parental support or virtual social interactions with internalizing symptoms differed by gender, nor did the longitudinal protective association of in‐person social interactions vary across gender groups. The one exception was a gender difference in the overall association between in‐person social interactions and internalizing symptoms, such that greater in‐person social interaction was associated with lower internalizing symptoms among boys but not girls. However, we found no evidence that the time‐varying association between in‐person social interactions and internalizing symptoms differed by gender. Taken together, these findings suggest that girls' elevated risk for internalizing difficulties during the pandemic may be explained by broader gender‐related vulnerabilities rather than differential effects of parental support or social interactions.

Youth who reported higher parental support at the initial timepoint had lower internalizing symptoms at baseline, and this difference was maintained across subsequent waves. This finding extends prior cross‐sectional and pre‐pandemic research by demonstrating that the protective role of parental support conferred an early advantage that was sustained beyond the early waves of the pandemic, and remained lower relative to their peers across all waves, even as external stressors evolved (Park et al. 2023). Supportive caregiving may promote emotion regulation, security, or adaptive coping, thus helping youth adjustment regardless of continued uncertainty at a societal level. From a policy perspective, these results underscore the likely value of family‐based supports during prolonged crises.

In contrast, the association between in‐person social interactions and internalizing symptoms was time‐dependent: Early in the study period, when public health restrictions were more stringent, in‐person contact was unrelated to internalizing symptoms. However, this association became increasingly negative over time, such that more frequent in‐person interactions was linked with lower internalizing symptoms at later waves, that is, as restrictions eased, and particularly for boys. This pattern suggests that opportunities for face‐to‐face interactions may have played a critical role in emotional recovery as the pandemic progressed, highlighting the growing importance of in‐person peer engagement as societal restrictions eased, communities re‐opened, and social opportunities re‐emerged.

In contrast, virtual interactions were not associated with internalizing symptoms, nor did their association change over time. Whereas virtual contact may have served an important role in maintaining social connection during periods of restriction, these connections did not show the same strengthening association with internalizing symptoms over time as observed for in‐person interactions. This finding contrasts some early pandemic studies that indicated virtual interactions were associated with lower loneliness and internalizing symptoms among youth (Marciano et al. 2022). However, the results align with literature with emerging adults that suggests virtual interactions were only weakly related (or unrelated) to momentary and long‐term well‐being during the pandemic, even when including additional modalities such as voice or text communication (Liang et al. 2024; Monninger et al. 2023). Importantly, virtual interactions were also not associated with *greater* internalizing symptoms, suggesting that this form of communication was also not harmful for this aspect of youth mental health, but instead may have been insufficient to fully substitute for in‐person social experiences. It is possible that the limited effects for virtual interactions reflect the lack of shared activities, nonverbal cues, and physiological co‐regulation characteristic of in‐person interactions.

Finally, the effect of in‐person interactions did not differ with baseline parental support. Instead, parental support and in‐person social interactions appeared to operate as independent, complementary protective factors. These findings suggest that supportive family environments did not offset the benefits of peer interaction during the pandemic, nor did peer interactions compensate for low parental support. Rather, both contexts appeared to contribute uniquely to youth emotional adjustment, underscoring the importance of supporting adolescents across multiple relationship domains. Importantly, though, as parental support was measured only at baseline, these findings should be interpreted as reflecting between‐family differences in perceived support, rather than changes in family relationships during the pandemic or over time.

### 4.1 Strengths and Limitations

This study had several strengths, including the population‐based sample, longitudinal design spanning multiple pandemic phases, and repeated youth‐reported measures. Several limitations should also be noted. Parental support was assessed only at baseline, precluding examination of time‐varying changes in family relationships. Measures of social interaction captured frequency but not quality or content, which may demonstrate differential associations (Wang and Lee 2026). Additionally, unmeasured contextual and individual factors (e.g., local policy variations, access to mental health services, or identity‐related stressors) may have affected symptom trajectories. Further, study waves were intentionally aligned with different phases of pandemic restrictions, which increased the real‐world relevance of the design; however, because restriction phases and assessment occasions were closely linked, it was not possible to fully disentangle the effects of changing public health restrictions from broader temporal processes occurring across the pandemic. Although supplementary analyses examining nonlinear trends and alternative indicators of restriction severity did not suggest that restriction‐related measures explained symptom trajectories better than a continuous measure of time, these findings should nevertheless be interpreted cautiously. Future research may also examine bidirectional associations between parental support and youth mental health (Gouze et al. 2017), as well as potential time‐varying changes in family relationships and their interplay with relationships with peers, and explore potential mediating mechanisms, such as emotion regulation strategies or loneliness (Cohodes et al. 2025).

## 5 Conclusion

Across an extended period of pandemic‐related social disruption characterized by alternating periods of stricter and more relaxed public health restrictions, this study suggests small increases in internalizing symptoms beyond the initial phases of the pandemic; in particular, for girls and at the later pandemic waves. Baseline parental support (across waves) and in‐person social interactions (increasingly over time) emerged as complementary protective factors for internalizing concerns. These findings suggest that recovery planning for future crises should be inclusive of gender‐specific supports and prioritize both strengthening family relationships and rebuilding safe, meaningful opportunities for peer interaction. Investments that support youth across home and community contexts may yield sustained benefits for adolescent mental health during future periods of widespread disruption.

## Author Contributions

**Nicole S. J. Dryburgh:** conceptualization, investigation, writing – original draft, methodology, visualization, formal analysis. **Ryan Miller:** investigation, writing – review and editing. **Eric Duku:** investigation, writing – review and editing. **Katherine T. Cost:** writing – review and editing. **Yun‐Ju (Claire) Chen:** writing – review and editing, investigation. **Jordan Edwards:** writing – review and editing. **Emma Nolan:** writing – review and editing. **Katholiki Georgiades:** writing – review and editing, conceptualization, investigation, funding acquisition, methodology, supervision.

## Funding

Funding for this research was provided by the Ontario Ministry of Health.

## Ethics Statement

This study was approved by the Hamilton Integrated Research Ethics Board at McMaster University (HiREB Project #: 10855).

## Consent

Informed consent was obtained from all individual participants included in the study.

## Conflicts of Interest

The authors declare no conflicts of interest.

## Supporting information


Supporting File

## Data Availability

The data that support the findings of this study are available from the corresponding author upon reasonable request. The data, analytic code, and materials necessary to reproduce the analyses presented here are not publicly accessible but are available upon request.

## Footnotes

1 Of note, sensitivity analyses examining changes in internalizing symptoms over time using participants' (1) regional stringency index and (2) Public Health Unit lockdown status rather than time yielded similar conclusions and did not improve model fit. Results are available upon request.
