# Supplementary material for: Interpersonal Predictors of Youth Internalizing Symptom Trajectories During the COVID‐19 Pandemic
Source: J Adolesc. 2026 Aug 4;98(7):2628–37. doi: 10.1002/jad.70245 (PMC13643676; doi:10.1002/jad.70245)
Supplement: Supplementary file 1 [file JAD-98-2628-s001.docx]

**Supplementary Materials: Interpersonal Predictors of Youth Internalizing Symptom Trajectories during the COVID-19 Pandemic**

**Supplemental Table S1**

*Comparison of gender and age for full sample vs. analytical sample.*

|  | **Participating Youth**  **(n = 230)** | **Analytic Sample (n = 176)** |
| --- | --- | --- |
| **Child Gender, n (%)** |  |  |
| Boy | 45.2 | 43.8 |
| Girl | 50.0 | 51.1 |
| Gender minority | 4.3 | 4.5 |
| Mixed report | 0.4 | 0.6 |
| **Child Age, M (SD)** | 14.43 (2.08) | 14.53 (2.07) |
